# Combining antibiotic with anti-TLR2/TLR13 therapy prevents brain pathology in pneumococcal meningitis

**DOI:** 10.1172/jci.insight.165737

**Published:** 2024-02-15

**Authors:** Susanne Dyckhoff-Shen, Ilias Masouris, Heba Islam, Sven Hammerschmidt, Barbara Angele, Veena Marathe, Jan Buer, Stefanie Völk, Hans-Walter Pfister, Matthias Klein, Uwe Koedel, Carsten J. Kirschning

**Affiliations:** 1 Department of Neurology, LMU University Hospital, LMU Munich, Germany.; 2 Institute of Medical Microbiology, University Hospital Essen, University of Duisburg-Essen, Essen, Germany.; 3 Department of Molecular Genetics and Infection Biology, Interfaculty Institute for Genetics and Functional Genomics, University of Greifswald, Greifswald, Germany.

**Keywords:** Infectious disease, Bacterial infections, Cellular immune response, Innate immunity

## Abstract

Despite effective antibiotic therapy, brain-destructive inflammation often cannot be avoided in pneumococcal meningitis. The causative signals are mediated predominantly through TLR-recruited myeloid differentiation primary response adaptor 88 (MyD88), as indicated by a dramatic pneumococcal meningitis phenotype of *Myd88^–/–^* mice. Because lipoproteins and single-stranded RNA are crucial for recognition of Gram-positive bacteria such as *Streptococcus* *pneumoniae* by the host immune system, we comparatively analyzed the disease courses of *Myd88^–/–^* and *Tlr2^–/–^ Tlr13^–/–^* mice. Their phenotypic resemblance indicated TLR2 and -13 as master sensors of *S*. *pneumoniae* in the cerebrospinal fluid. A neutralizing anti-TLR2 antibody (T2.5) and chloroquine (CQ) — the latter applied here as an inhibitor of murine TLR13 and its human ortholog TLR8 — abrogated activation of murine and human primary immune cells exposed to antibiotic-treated *S*. *pneumoniae*. The inhibitory effect of the T2.5/CQ cocktail was stronger than that of dexamethasone, the current standard adjunctive drug for pneumococcal meningitis. Accordingly, TLR2/TLR13 blockade concomitant with ceftriaxone application significantly improved the clinical course of pneumococcal meningitis compared with treatment with ceftriaxone alone or in combination with dexamethasone. Our study indicates the importance of murine TLR13 and human TLR8, besides TLR2, in pneumococcal meningitis pathology, and suggests their blockade as a promising antibiotic therapy adjunct.

## Abstract 2

TLR2 and TLR13 play essential roles in experimental pneumococcal meningitis pathology, suggesting TLR2/TLR13 blockade as a promising adjunctive therapy for antibiotics.

## Graphical abstract

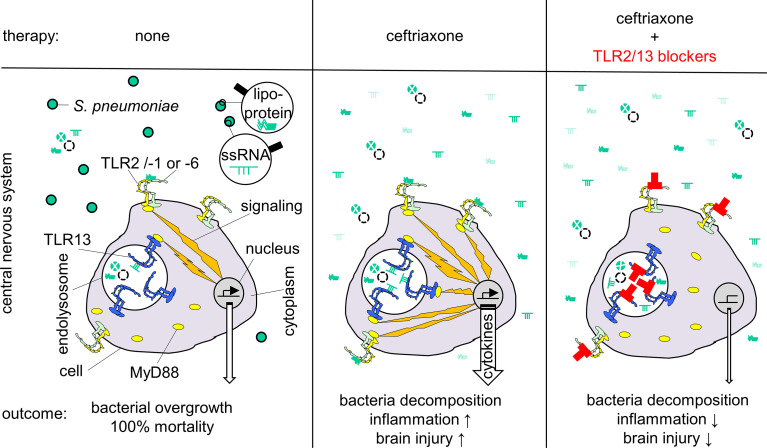

## Introduction

Although effective antibiotic therapy is available, pneumococcal meningitis remains a serious health threat, with death or neurologic sequelae in about half of cases in industrialized countries (1–3). Its poor prognosis is related to the inability of the host to successfully fight pathogens within the cerebrospinal fluid (CSF) space due to a lack of opsonins and phagocytes (2, 3). As a result, *Streptococcus pneumoniae* can multiply in the CSF to concentrations comparable to those achieved when cultured in specific growth media (4). Prompt antibiotic therapy with bacteriolytic β-lactams, the current gold standard for treating invasive pneumococcal infections, is thus key to saving life and function (5). Delaying antibiotic therapy, however, correlates with an increase in bacterial load and, as a result, the enlargement of the source of pneumococcal pathogen-associated molecular patterns (PAMPs) released into the CSF by β-lactam administration (6). High doses of PAMPs, recognized by pattern recognition receptors (PRRs), can hyperactivate resident cells, leading to a cytokine “storm” instead of an appropriate “wave.” This can, in turn, cause collateral neural damage, which is often irreversible (2, 3, 7). A more comprehensive understanding of how the immune system senses cerebral *S*. *pneumoniae* could guide effective therapeutic interventions to counteract the pathology associated with pneumococcal meningitis.

Mice lacking expression of myeloid differentiation primary response adaptor protein 88 (MyD88), which is recruited by 9 out of the 10 human and 11 out of the 12 murine Toll-like receptors (TLRs, besides IL-1 receptor I–type molecules), displayed massively reduced acute brain inflammation and pathology as compared with wild-type (WT) littermates (8). Thus, from within the family of PRRs, TLRs may dominate sensing of *S*. *pneumoniae* and innate immune activation inside the CSF (2, 9, 10). Previous analyses of mice lacking expression of single or multiple TLRs such as TLR2, -4, and/or -9 suggested involvement of all 3 in *S*. *pneumoniae*–induced immune cell activation in vitro as well as in pneumococcal pneumonia (11–13), but only of the first 2 in pneumococcal meningitis (14, 15). Although capable of modulating lung inflammation, singular TLR4 deficiency did not affect meningitis pathology (12–14). Strikingly, the meningitis phenotype of *Tlr2^–/–^ Tlr4^–/–^* (*Tlr2/4^–/–^*) double-knockout (double-KO) mice was stronger than that of *Tlr2^–/–^* mice, yet significantly milder than that of *Myd88^–/–^* mice. The exceptional strength of the latter phenotype implies involvement of further TLR activity beyond that of the TLRs mentioned so far in pneumococcal meningitis (2, 14). Upon intranasal pneumococcal infection, *Tlr7/9/13^–/–^* mice (but not the respective single-KO strains) displayed a *Myd88^–/–^*-like phenotype and rapidly succumbed to infection (16). Correspondingly, full cytokine production upon pneumococcal exposure required concerted expression of TLR7, -9, and -13 in alveolar macrophages and microglial cells, but not in peritoneal macrophages (16). Accordingly, while Myd88 deficiency resulted in markedly reduced purulent meningitis compared with WT mice, *Myd88^–/–^* mice exhibited an even more pronounced pneumonia secondary to meningitis than their corresponding WT counterparts (8). Taken together, the data suggest that the PRR repertoire involved in *S*. *pneumoniae* detection varies among cells and tissues, conceptually affecting therapeutic PRR targeting in distinct pneumococcal infections.

With the goal of identifying the PRRs crucial for sensing pneumococci within the CSF, and considering specific TLRs as prime candidates, we subjected specific TLR-KO mouse strains to experimental pneumococcal meningitis. As a result, we pinpointed the key cerebral PRRs responsible for detecting pneumococcal infections and associated pathology to a specific TLR pair. More precisely, TLR2 and -13 emerged as the primary sensors for detecting pneumococcal infections in murine CSF. When we combined antibiotic treatment with dual antagonism of TLR2 and -13, we found that this approach provided more effective protection against pneumococcal meningitis pathology in WT mice compared with the combination of antibiotics and the gold standard dexamethasone (1–3). Consequently, the simultaneous inhibition of TLR2 and -8, the human counterpart of TLR13, in combination with ceftriaxone administration, whether with or without dexamethasone, significantly suppressed the activation of peripheral blood mononuclear cells (PBMCs) and — albeit to a smaller extent — whole blood cells, when exposed to *S*. *pneumoniae*. This suggests that both receptors hold promise as molecular targets for adjunctive antibiotic therapy in the treatment of pneumococcal meningitis.

## Results

### Endosomal TLR function is critically involved in innate immunity to pneumococcal infection of the CNS.

**Figure 1:**
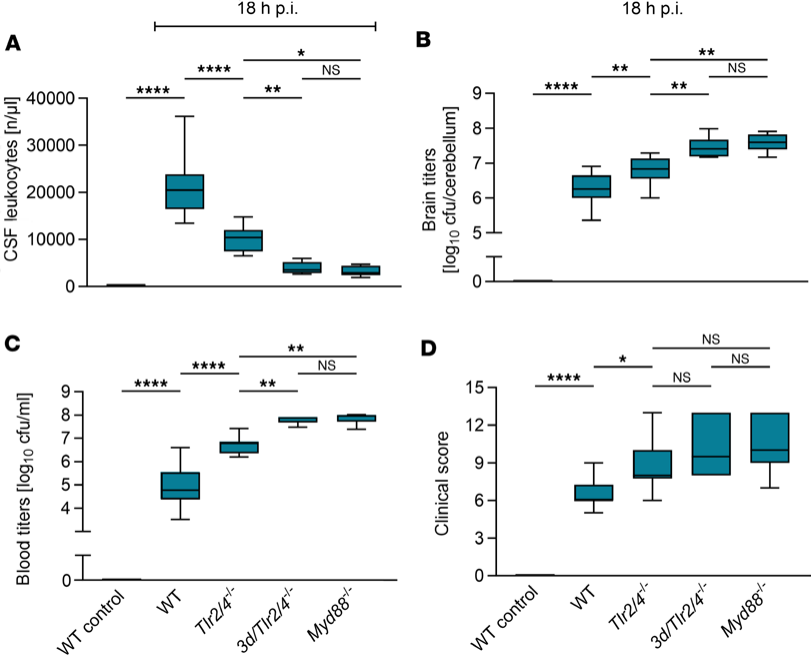
Strong pneumococcal meningitis phenotypes of *3d/Tlr2/4^–/–^* and *Myd88^–/–^* mice are largely indistinguishable. (**A**–**D**) Pneumococcal meningitis was elicited by intracisternal injection of 1 × 10^5^ CFU viable *Streptococcus* *pneumoniae*. Eighteen hours postinfection (p.i.), mice of the indicated genotypes (WT, *n* = 18; *Tlr2/4^–/–^*, *n* = 10; *3d/Tlr2/4^–/–^*, *n* = 10; *Myd88^–/–^*, *n* = 9; negative control with PBS, *n* = 8) were analyzed. After clinical examination (including the collection of clinical score values), CSF was obtained to determine leukocyte counts (**A**). Subsequently, the mice were sacrificed, and cerebellum (**B**) and blood (**C**) samples were collected for bacterial titer determinations. (**D**) Clinical scores for the mice analyzed in **A**–**C**. Data are presented as median (line in box), 25%–75% percentile range (bounds of the box), minimum and maximum (whiskers). The number of samples in individual experiments may be lower if mice had to be euthanized before the end of the experiment or technical issues appeared (for detailed information see Supporting Data Values file). Statistical test was 1-way ANOVA and Tukey’s post hoc test. ****P < 0.0001; **P < 0.01; *P < 0.05. NS, not significant; CFU, colony-forming units.

We previously implicated the concerted action of TLR2 and -4 as a driver of hyperinflammation in murine *S*. *pneumoniae* meningitis (14). Strikingly, *Tlr2/4^–/–^* mice by far failed to equal the strong disease phenotype of *Myd88^–/–^* mice (8). We therefore hypothesized that one or more additional TLRs may contribute to the MyD88 deficiency phenotype and aimed at the identification of the minimally multiple TLR-deficient mouse strain whose phenotype would most closely resemble that of *Myd88^–/–^* mice. First, we comparatively monitored the course of experimental pneumococcal meningitis in WT, *Tlr2/4^–/–^*, *Myd88^–/–^*, and *3d/Tlr2/4^–/–^* mice. The latter group, apart from lacking TLR2 and -4 expression, exhibits no endosomal TLR activity due to expression of an inactive Unc93b1 (*3d*) variant (17–19). While CSF leukocyte counts were reduced by 50% in *Tlr2/4^–/–^* mice, paralleled by significant increases in brain and blood bacterial loads, as compared with WT mice (Figure 1), immune dysfunction in *3d/Tlr2/4^–/–^* mice was significantly more pronounced and equaled that of *Myd88^–/–^* mice. Specifically, *3d/Tlr2/4^–/–^* and *Myd88^–/–^* mice exhibited a similar reduction in CSF pleocytosis by 82% and 84%, respectively, as compared with WT mice (Figure 1). The marked weakness of the immune response in both KO strains was mirrored by a 13-fold and 16-fold rise in brain bacterial loads (*3d/Tlr2/4^–/–^* and *Myd88^–/–^*, respectively), and worsening of disease, represented by increased clinical score values as compared with infected WT mice (Figure 1; graphs with complete-integer *y* axes representing bacterial loads are shown in Supplemental Figure 1; supplemental material available online with this article; https://doi.org/10.1172/jci.insight.165737DS1). This result implicated a substantial involvement of a nucleic acid–sensing endosomal TLR activity in pneumococcal meningitis.

### TLR13 and TLR2 are major pneumococcal sensors in the subarachnoid space.

**Figure 2:**
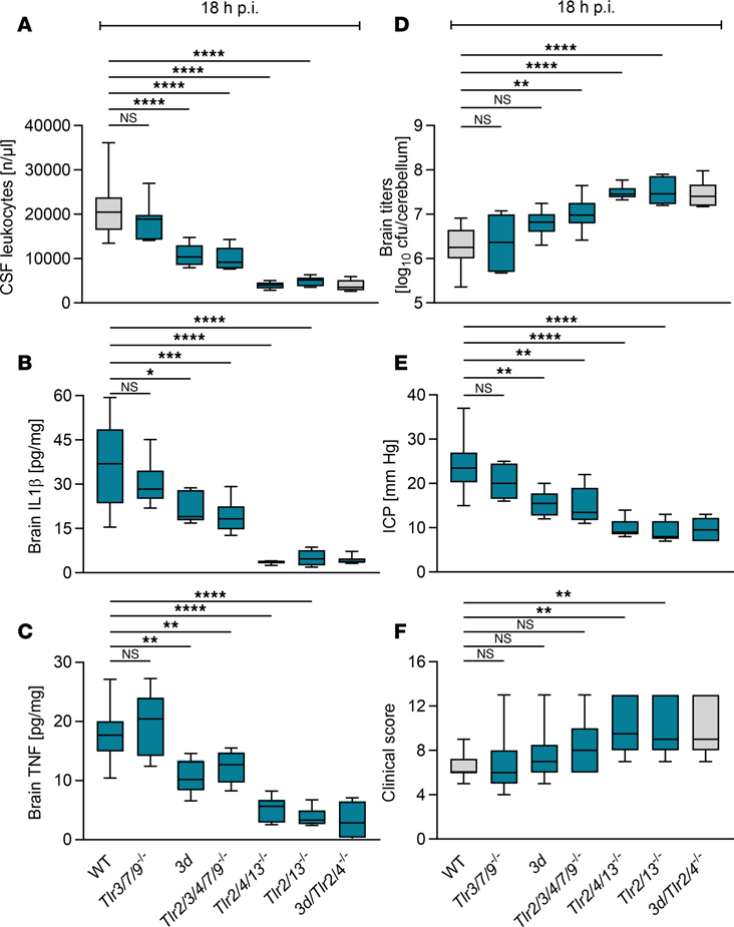
TLR13 mediates pathogen recognition in addition to TLR2 in murine pneumococcal meningitis. (**A**–**F**) Pneumococcal meningitis was modeled as specified for Figure 1 in mice of the 7 genotypes indicated (WT, *n* = 18; *Tlr3/7/9^–/–^*, *n* = 9; *3d*, *n* = 9; *Tlr2/3/4/7/9^–/–^*, *n* = 10; *Tlr2/4/13^–/–^*, *n* = 10; *Tlr2/13^–/–^*, *n* = 10; *3d/Tlr2/4^–/–^*, *n* = 10). CSF leukocyte counts (**A**), brain IL-1β (**B**) and TNF levels (**C**), brain bacterial loads (**D**), ICP (**E**), and clinical score values (**F**) were measured. Data are presented as median (line within box), 25%–75% percentile range (bounds of the box), and minimum and maximum (whiskers). The data from the WT and *3d/Tlr2/4^–/–^* groups (boxes shown in gray) correspond to those in Figure 1. The number of samples in individual experiments may be lower if mice had to be euthanized before the end of the experiment or technical issues appeared (for detailed information see Supporting Data Values file). Statistical test was 1-way ANOVA and subsequent Tukey’s post hoc test. ****P < 0.0001; ***P < 0.001; **P < 0.01; *P < 0.05, as compared with infected WT mice–derived samples. NS, not significant.

In an attempt to comprehensively analyze endosomal TLR activity, we compared *Tlr3/7/9^–/–^*, *3d*, *Tlr2/3/4/7/9^–/–^*, *Tlr2/4/13^–/–^*, and *Tlr2/13^–/–^* with WT and *3d/Tlr2/4^–/–^* mouse disease phenotypes (20). Except for *Tlr3/7/9^–/–^* mice, the immune responses of all the mutant mouse strains were significantly impaired, as indicated by reduced CSF leukocyte counts, decreased brain IL-1β and TNF levels, increased bacterial outgrowth, and an aggravated disease (Figure 2). The phenotypes of *Tlr2/4/13^–/–^* and *Tlr2/13^–/–^* mice were notably fulminant and nearly indistinguishable from the phenotype of their *3d/Tlr2/4^–/–^* counterparts. For instance, CSF leukocyte numbers were 81%, 77%, and 82% lower, whereas brain bacterial loads were increased 12-fold, 15-fold, and 13-fold and blood loads increased 84-fold, 63-fold, and 91-fold, respectively (Figure 2 and Supplemental Figure 1, right panel, the ordinate values comprehensively span the range from 0 to 10 log_10_ CFU per compartment or volume), as compared with infected WT mice. These changes were paralleled by a worsening of disease as compared with infected WT mice, indicated by mostly significantly higher clinical score values. The more severe disease course is likely attributable to more severe bacteremia (Figure 2 and Supplemental Figure 1, right panel) and aggravation of systemic complications, as we previously observed in *Myd88^–/–^* mice (8) because intracranial complications were equally and significantly attenuated in *Tlr2/4/13^–/–^*, *Tlr2/13^–/–^*, and *3d/Tlr2/4^–/–^* as compared with WT mice.

The disease phenotypes of *3d* and *Tlr2/3/4/7/9^–/–^* mouse strains were substantially less pronounced (Figure 2). For instance, in mice of these 2 strains CSF leukocyte numbers were reduced by approximately 50%, resembling the *Tlr2/4^–/–^* mouse phenotype.

The indistinguishable disease phenotypes of *Tlr2/13^–/–^* and *Tlr2/4/13^–/–^* mice, along with their similarity to the *Myd88^–/–^* disease phenotype, suggest that TLR2 and -13 are the primary drivers of innate immune activation during pneumococcal infection of the murine CNS. This aligns with our earlier findings implicating both receptors in the sensing of Gram-positive bacteria (20).

### Dual antagonism of specific TLR activities is a substantially more potent adjunct to antimicrobial therapy of murine pneumococcal meningitis than dexamethasone.

**Figure 3:**
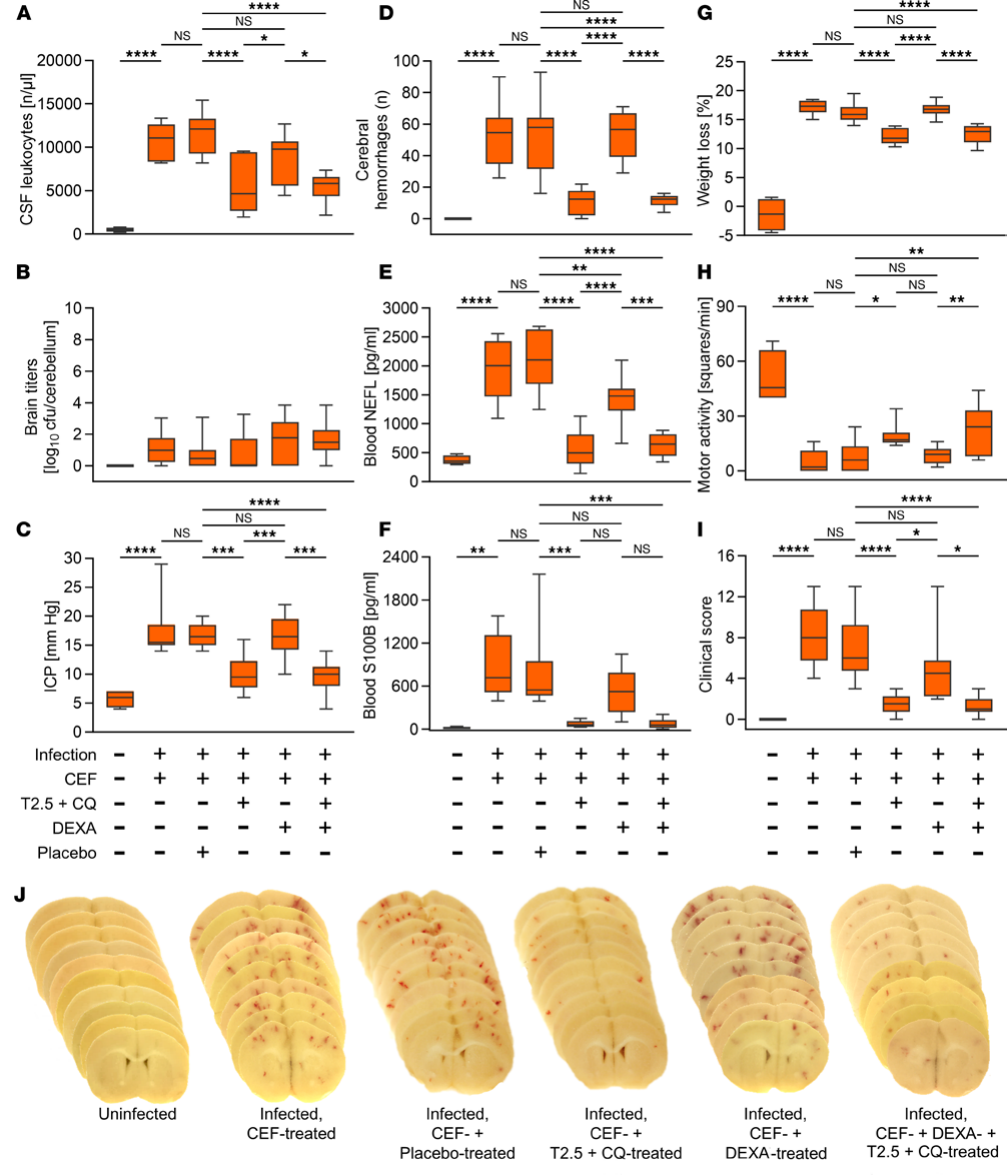
The adjunctive use of neutralizing anti-TLR2 monoclonal antibody and chloroquine in antibiotic therapy provides significantly greater benefits to mice with pneumococcal meningitis as compared with administration of dexamethasone. (**A**–**J**) Pneumococcal meningitis was elicited by intracisternal injection of viable *Streptococcus* *pneumoniae* (with PBS as negative control, *n* = 4). Eighteen hours postinfection (p.i.), animals were treated with ceftriaxone (*n* = 10) and a cocktail of neutralizing anti-TLR2 mAb (T2.5) and chloroquine (CQ) (*n* = 10), with the additional (*n* = 10) or alternative (*n* = 12) administration of dexamethasone (DEXA) or placebo (*n* = 14). At 42 hours p.i., CSF leukocyte counts (**A**), bacterial load (**B**), intracranial pressure (ICP) (**C**), the number of cerebral hemorrhages (**D**), as well as plasma concentrations of neurofilament light chain (NEFL) (**E**) and S100B (**F**) — 2 biomarkers for brain injury — were evaluated. Mice also underwent clinical examination, including the assessment of weight loss (**G**), motor activity (**H**), and clinical scores (**I**). (**J**) Brain sections from representative mice of the investigated groups. Data are presented as median (line in box), 25%–75% percentile range (bounds of the box), and minimum and maximum (whiskers). The number of samples in individual experiments may be lower if mice had to be euthanized before the end of the experiment or technical issues appeared (for detailed information see Supporting Data Values file). Statistical test was 1-way ANOVA and subsequent Tukey’s post hoc test. ****P < 0.0001; ***P < 0.001; **P < 0.01; *P < 0.05. NS, not significant; CEF, ceftriaxone.

A combination of antimicrobial and immunosuppressive therapy is crucial for improving the prognosis of pneumococcal meningitis (2, 3). Dexamethasone is currently the only adjunctive drug approved for treatment of adults suffering from pneumococcal meningitis. Adjunctive dexamethasone halves mortality, yet largely fails to prevent neurological complications (3, 21, 22). Given the apparent need for more potent adjunctive treatments (23), we investigated whether mice with antibiotic-treated pneumococcal meningitis would benefit from concurrent TLR2 and -13 blockade. At 18 hours postinfection (p.i.), mice were concurrently treated with ceftriaxone, the antagonistic monoclonal anti-TLR2 antibody T2.5 (of which a humanized surrogate has been derived and clinically established; refs. 24–26), and the endosomal TLR inhibitor chloroquine (CQ), a quinine-based antimalarial drug established for 80 years (27, 28). The treatment’s effectiveness was assessed 24 hours later. Adjunctive dual TLR blockade resulted in a significant reduction in CSF pleocytosis, whereas pneumococcal elimination remained unaffected (Figure 3). The reduction in inflammation was associated with significantly lower intracranial pressure (ICP) values and fewer hemorrhagic spots in the brains of mice treated with the dual TLR inhibitor cocktail and ceftriaxone. This pattern held true for plasma concentrations of 2 well-established neuronal injury biomarkers, namely NEFL and S100B (29–32). This observation remained consistent regardless of whether mice received additional dexamethasone treatment or not. In contrast, groups treated with ceftriaxone only, placebo, and the combination of dexamethasone and ceftriaxone showed worse outcomes (Figure 3). Correlating with a reduction in neurological complications, adjunctive dual TLR activity blockade led to an improved clinical outcome in experimental pneumococcal meningitis as compared with placebo treatment. Animals subjected to the anti-TLR treatment regimen showed less pronounced hypothermia, loss of body weight, and reduced motor activity, as well as lower clinical scores compared with the mice in the latter group (Figure 3 and Supplemental Figure 2). Next, we asked whether adjunctive dual TLR inhibitor cocktail administration remains effective when given after the start of antibiotic therapy. Thus, the T2.5 and CQ mix was administered to infected mice 3 hours after ceftriaxone had been applied. In this postantibiotic treatment scenario, a beneficial effect of adjunctive dual TLR blockade was not observable (e.g., CSF leukocytes: 12,175 ± 4,373 vs. 11,579 ± 2,420 cells/μL; ICP: 16.0 ± 3.4 vs. 16.7 ± 1.9 mmHg; and clinical score values: 5.0 ± 0.8 vs. 7.1 ± 3.3 in posttreated vs. placebo-treated mice), suggesting that TLR blockade prior to or synchronously with bacterial cell disintegration provides a therapeutic opportunity, while later the therapeutic window closes. We next compared the therapeutic efficacies of dexamethasone and the dual TLR blocker mix again in the simultaneous ceftriaxone and adjunct application setting. The efficacy of T2.5 and CQ was superior to that of dexamethasone, and upon coadministration with dexamethasone, the beneficial effect of the anti-TLR cocktail was not compromised (see Figure 3). Specifically, CSF pleocytosis, the rise in ICP, the extent of cerebral bleeding, the increase in plasma NEFL and S100B levels, as well as clinical symptoms, were significantly reduced in infected mice that received combination therapy with ceftriaxone, T2.5, and CQ, regardless of whether dexamethasone was coadministered or not. This reduction was observed in comparison with mice with meningitis treated solely with ceftriaxone, as well as those given a placebo or dexamethasone in combination with ceftriaxone.

The pneumococcal meningitis model we employed, through intracisternal (i.c.) inoculation, extends beyond the CNS compartment in certain aspects. Pneumococci can traverse the blood-brain barrier, moving from CSF into the bloodstream within a few hours, allowing dissemination to and infection of other organs such as the lungs. However, it is unlikely that septic and pulmonary complications played a decisive role in our experimental approaches. The experiments were either terminated quite early (18 hours p.i.), or a highly effective antimicrobial treatment was initiated at an early stage (18 hours p.i.), preventing the manifestation of these complications. To provide an initial indication of whether our observations are transferable to other (primarily systemic) pneumococcal disease models, we conducted additional experiments using an intravenous (i.v.) challenge model. In this model, the TLR cocktail was administered to mice 1 hour before i.v. injection with 1 × 10^8^ CFU of ceftriaxone-killed *S*. *pneumoniae*, while the control group received PBS treatment. Specimens were collected at both 2-hour and 6-hour time points after bacterial challenge, including blood plasma, spleen, and lung homogenates. Under the selected conditions, the 2-hour samples displayed substantial immune responses in all examined tissues (such as increased expression of IL-1β, IL-6, or TNF; Supplemental Figure 3). However, by the 6-hour time point, visible immune activity had significantly diminished. At both time points, virtually no distinction between the untreated group and the group treated with the dual blocker cocktail was observable, indicating that the treatment was ineffective in inhibiting the immune response triggered by systemically applied antibiotic-killed *S*. *pneumoniae* in the examined compartments. This negative result suggests that it would not be appropriate to extrapolate our findings from experimental pneumococcal meningitis to other pneumococcal diseases. Collectively, our findings indicate dual TLR blockade as a very promising approach, specifically for adjunctive pneumococcal meningitis therapy, which neither affects nor is affected by dexamethasone therapeutic activity.

### Dual TLR antagonism abrogates murine and human primary immune cell activation upon exposure to viable S. pneumoniae and subsequent ceftriaxone application.

**Figure 4:**
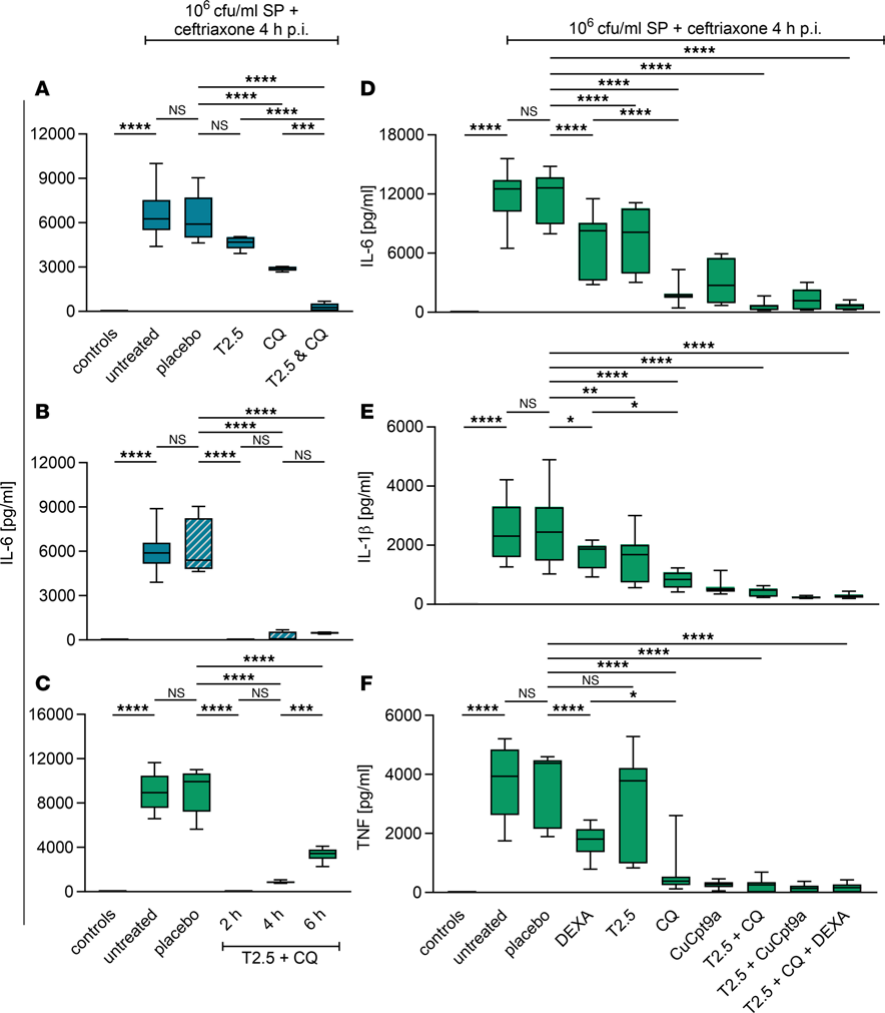
Dual neutralizing TLR2 mAb and CQ application in vitro abrogates *Streptococcus* *pneumoniae*–induced cytokine release by murine and human immune cells. (**A** and **B**) Murine BMDMs (blue boxes) and (**C**–**F**) human PBMCs (green boxes) were cultured and challenged with 1 × 10^6^ CFU/mL viable *S*. *pneumoniae* (SP, except for controls, which received THY solution only). Four hours postinfection (p.i.), cells were treated with ceftriaxone. Two hours earlier (2 h), simultaneously (4 h), or 2 hours later (6 h p.i.), anti-TLR2 mAb (T2.5) and chloroquine (CQ) were added to cell cultures. In human PBMC cultures, the TLR8 inhibitor Cu-Cpt9a and dexamethasone (DEXA) were applied additionally where indicated. Cell culture supernatants were sampled 24 hours p.i., aliquoted, and stored until ELISA analysis. Supernatants were analyzed for IL-6 (**A**–**D**), IL-1β (**E**), and TNF (**F**). The experiments with BMDMs were conducted at least 2 times in triplicate, while those involving human PBMCs were performed at least 2 times in quadruplicate (for detailed information see Supporting Data Values file). The data for the placebo and T2.5 plus CQ (4 h) groups in **A** and **B** (as indicated by blue boxes with gray diagonal stripes in **B**) largely overlap. Data are depicted as median (line in box), 25%–75% percentile range (bounds of the box), and minimum and maximum (whiskers). Statistical test was 1-way ANOVA and subsequent Tukey’s post hoc test. ****P < 0.0001; ***P < 0.001; **P < 0.01; *P < 0.05. NS, not significant.

Lack of TLR13 and functional TLR8 expression distinguishes humans and other primates from mice and numerous other animals (33). Aiming at the expansion of analysis from mice to a human experimental system, we applied the dual anti-TLR treatment side by side to murine bone marrow–derived macrophages (BMDMs) and human PBMCs as surrogates of both species’ innate immune systems. Four hours after challenge with viable *S*. *pneumoniae*, both kinds of immune cell cultures were treated with ceftriaxone; a T2.5 and CQ mix was applied 2 hours prior, simultaneously with, or 2 hours after ceftriaxone administration. Dual TLR blockade completely abrogated IL-6 production by pneumococci-challenged murine macrophages, irrespective of the timely order of the treatments (Figure 4). Similarly, human PBMCs largely failed to respond to *S*. *pneumoniae* exposure, especially upon pre- and cotreatment with dual TLR inhibitors. Adjunctive treatment either with T2.5 or CQ alone was significantly less effective as compared with the adjunctive application of both TLR inhibitors together (Figure 4). Next, we mirrored dual TLR blockade against dexamethasone. Dexamethasone was equally effective as exclusive TLR2 blockade by T2.5, but significantly less effective than CQ application, and even less effective than T2.5 plus CQ coapplication (Figure 4). Notably, dexamethasone did not interfere with the activities of dual TLR blockade and vice versa.

**Figure 5:**
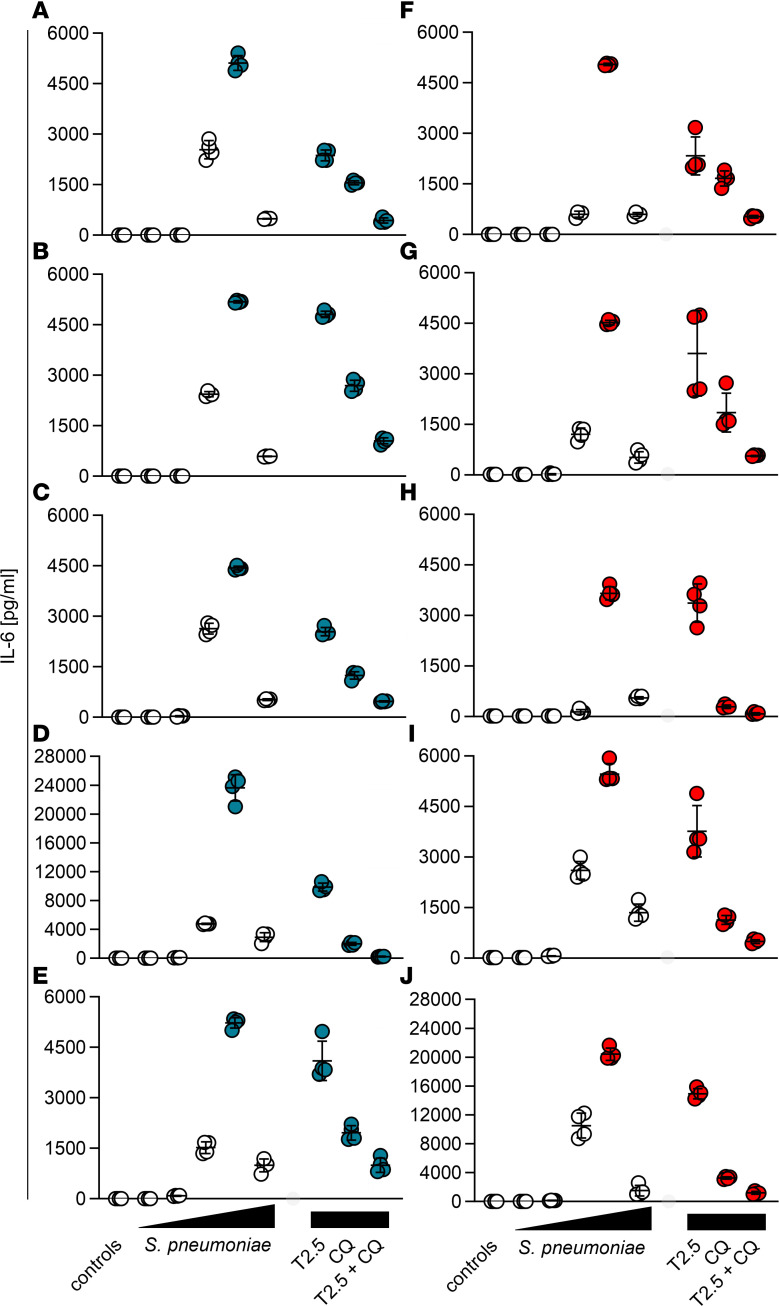
Dual TLR inhibitor cocktail application inhibits in vitro antibiotic–treated *Streptococcus* *pneumoniae* infection–induced IL-6 release by PBMCs donated by 10 human healthy volunteers. (**A**–**J**) Cultures of peripheral blood mononuclear cells (PBMCs) from 10 healthy human individuals were challenged with increasing doses of viable *S*. *pneumoniae* (triangles, sequentially 1 × 10^3^, 1 × 10^4^, 1 × 10^5^, 1 × 10^6^, and 1 × 10^7^ CFU/mL; controls received a sheer sterile THY solution). At the 4-hour postinfection (p.i.) time point, ceftriaxone was applied to all cultures, while anti-TLR2 mAb (T2.5) and/or chloroquine (CQ) were specifically added where indicated (rectangles, 1 × 10^6^ CFU/mL). Supernatants were harvested 24 hours p.i. and analyzed by ELISA. Blue, male donors (**A**–**E**); red, female donors (**F**–**J**); red and blue data points indicate challenges with 1 × 10^6^ CFU/mL *S*. *pneumoniae*. The results are from 2 independent experiments with duplicates, presented as individual dots with mean ± SD.

CQ is known to inhibit all nucleic acid–sensing endosomal TLRs; we supposed dominance of TLR8 in human cellular *S*. *pneumoniae* sensing. Therefore, alternatively to CQ, we applied the TLR8-specific inhibitor Cu-Cpt9a to evaluate our assumption (33). Cu-Cpt9a inhibited *S*. *pneumonia*e–induced human PBMC activation similarly effectively as CQ, and replacement of CQ with Cu-Cpt9a in the dual TLR blocker mix did not result in a loss of effectiveness. Both observations indicate TLR8 as the CQ target in this experimental setting (Figure 4). In order to accommodate stronger genetic diversity of the clinic as compared with mouse experimentation, additional analyses of PBMCs isolated from 10 healthy individuals (young adults, *n* = 5 of each sex) were performed, and dose-dependent IL-6 production upon pneumococcal challenge with reduced IL-6 release at the highest concentration due to increased cell death was detected. Furthermore, unanimously near-complete abrogation of IL-6 release upon cultural infections with *S*. *pneumoniae* (by 90.2% in the median, minimum 79.8%, maximum 99%) by dual TLR blockade was observed (Figure 5). Notably, the efficacies of T2.5 and CQ, when applied singularly, varied substantially between the individual specimens, yet in a sex-independent fashion. While T2.5 administration reduced *S*. *pneumoniae*–induced IL-6 release by a median of 30%, CQ inhibited IL-6 production by 70% (range, 48%–92%). These results suggest the predominant involvement of endosomal murine TLR13 and human TLR8, alongside TLR2, in *S*. *pneumoniae* sensing by first-line immune cells in both species. Consequently, expanding treatment with TLR2 and -8 blockade to meningitis patients at the initiation of antibiotic therapy — ideally as quickly as possible upon suspected diagnosis — may be a promising therapeutic approach.

Broadening our perspective with assays that encompass the circulatory compartment in its complexity, involving neutrophils and most serum components, we conducted experiments using whole-blood assays from healthy human volunteers. In these experiments, we assessed the effects of the dual TLR cocktail by comparing 2 different approaches with varying challenge protocols. In one approach, we preblocked whole-blood aliquots with the dual TLR cocktail, challenged them with ceftriaxone-killed pneumococci, and analyzed supernatants 6 hours later. In the other approach, we infected whole-blood samples with pneumococci and, 4 hours later, applied the TLR blocker cocktail along with ceftriaxone to analyze plasma supernatants 24 hours thereafter. In general, the “therapeutic” effects were significantly less pronounced compared with experiments using human PBMCs. While no significant changes in IL-6 concentrations were observed, the dual blocker cocktail demonstrated significant effects on IL-1β and TNF levels in both protocols (Supplemental Figure 4). The inhibitory capacity of the blocker cocktail appears largely attributable to CQ, as TLR2 blockade alone had no visible effect in either condition. Thus, the findings from the whole-blood assays suggest that the TLR cocktail may also exert an antiinflammatory effect on systemic pneumococcal infection, emphasizing the relevance of further investigations in other pneumococcal infection models.

## Discussion

Pneumococcal infection of the CNS generates some of the most powerful inflammatory responses known in medicine and is fatal if left untreated with antibiotics (34). By damaging host brain tissue collaterally, the inflammatory immune response can cause meningitis-associated brain damage and thus unfavorable disease outcomes (2, 3, 34). Initial innate immune activity is triggered by specific pneumococcal PAMPs binding to a previously elusive set of different signal-transducing PRRs expressed by resident cells. An abrupt release of large amounts of PAMPs into the CSF, such as upon bacteriolytic antimicrobial treatment (e.g., with ceftriaxone), can dramatically amplify immune activity, leading to a devastating cytokine storm (2). The robust acute meningitis phenotype observed in Myd88-deficient mice, characterized by an impaired host immune response and reduced brain pathology compared with infected WT mice, suggests that TLRs play a crucial role in eliciting the immune response to pneumococcal CNS infection. However, their involvement in the antibiotic-triggered inflammatory burst remains unclear (8, 14, 35). Yet, TLR2 has been implicated in sensing of pneumococcal components such as lipoprotein and endopeptidase O in vitro (36–38). However, the mild *Tlr2^–/–^* disease phenotype, as compared with its fulminant *MyD88^–/–^* counterpart, indicated involvement of additional TLR activity in meningeal pneumococcal recognition (8, 39). TLR4 has been attributed a role as a pneumococcal pneumolysin sensor (40), although this conclusion was later disputed (41). Furthermore, binding of pneumococcal DNA to TLR9 (42) and markedly attenuated responsiveness of *Tlr2/4/9^–/–^* macrophages to *S*. *pneumoniae* as compared with that of their single-KO counterparts have been reported (14, 43). Subsequent comparative analyses in an experimental meningitis model, however, implicated the concerted action of TLR2 and TLR4, while negating a role for TLR9 in the innate immunological perception of pneumococcal CNS infection (14, 15, 39, 44). Consistently, in our study, *Tlr2/4^–/–^* and *Tlr2/3/4/7/9^–/–^* mice displayed comparable phenotypes, while the phenotype of *Tlr3/7/9^–/–^* mice was largely indistinguishable from that of WT mice, arguing against a key role of TLR3, -7, and -9 in pneumococci sensing and thus meningitis pathology.

In contrast, *3d*/*Tlr2/4^–/–^*, *Tlr2/4/13^–/–^*, and *Tlr2/13^–/–^* mice displayed very similar phenotypes, virtually identical to the phenotype of *MyD88^–/–^* mice. This negates a central role for TLR4 involvement, while indicating that TLR2 and -13 form the major cellular sensor duplex for *S*. *pneumoniae* in the CSF. This result aligns with the fundamental role of TLR13-mediated recognition of a specific single-stranded RNA (ssRNA) segment harbored by clinically relevant bacterial 23S rRNA, which indicates, besides TLR2 ligands, presence of Gram-positive bacteria to the murine host (45–50). However, while increased susceptibility of *Tlr13^–/–^* mice to experimental pneumococcal encephalitis and decreased capability of TLR13-deficient microglia to respond to pneumococcal exposure have been reported, a comprehensive characterization of TLR involvement in the perception of pneumococcal CNS infection and the development of meningitis pathology remained pending (16).

In contrast to the exceptionally high demand of murine TLR13 for integrity of a relatively large 23S ssRNA segment and the involvement of murine TLR7 in bacterial tRNA sensing (20, 33, 51–53), human immune cells employ TLR7 and -8 for ssRNA sensing, with the latter of the 2 in a largely uracil- and RNase T2–dependent manner (54, 55). Moreover, total bacterial RNA such as that of *Borrelia burgdorferi* is sensed by human immune cells mainly through TLR8 (56). In addition to bacterial 23S rRNA, 16S and 5S bacterial rRNA activate human monocytoid THP1 cells through TLR8 (33, 57, 58). Along this line, exposure to *Staphylococcus aureus*, *Streptococcus* *pyogenes*, *S*. *pneumoniae*, *Streptococcus* *agalactiae*, and *Listeria monocytogenes* activated human monocytes in a TLR8-dependent manner, as TLR8 blockade inhibited cellular activations (59–64). Mutational *Tlr8* segment deletion in animals such as mice rendered TLR8 largely nonfunctional, which might have led to constitutive rodent and Lagomorpha expression of evolutionarily older TLR13 (33, 65). To confirm the previous identification of human TLR8 as a sensor of major Gram-positive bacteria and therein RNA, we side-by-side applied CQ as pan-endosomal TLR inhibitor and the selective TLR8 inhibitor Cu-Cpt9a (66) to human PBMCs undergoing pneumococcal infection in the absence or presence of TLR2-neutralizing T2.5. Indistinguishable inhibition potency of both CQ and Cu-Cpt9a under both conditions confirmed the conceptual role of TLR8 as a TLR13 successor and major human *S*. *pneumoniae* RNA sensor molecule.

Systemically, however, other PRRs beyond TLR2, -8, or -13, and even other endosomal TLRs, might also be involved in sensing *S*. *pneumoniae*. This conclusion is drawn from the fact that the same adjunctive treatment applied to mice with meningitis, specifically the T2.5 plus CQ cocktail, did not visibly affect short-lived inflammation upon systemic challenge with ceftriaxone-killed *S*. *pneumoniae*. The lack of therapeutic efficacy could be attributed to the stimulation model used, namely to the direct instillation of ceftriaxone-killed *S*. *pneumoniae* into the bloodstream. The RNA released during in vitro–induced bacterial lysis may undergo rapid degradation and inactivation by RNases present in the blood, thus potentially preventing its interaction with endosomal TLRs. Furthermore, it should be noted that viable *S*. *pneumoniae* can enter tissues such as the spleen from the bloodstream and be internalized by local macrophages (67), potentially facilitating the uptake of bacterial RNA by endosomes and consequent TLR13 activation. In addition, unlike the distinct outcomes observed in PBMC experiments, adjunctive treatment with the T2.5 plus CQ cocktail only mildly and selectively inhibited human whole-blood immune response during ex vivo pneumococcal infection. While no significant changes in IL-6 concentration were observed, the dual blocker cocktail significantly influenced IL-1β and TNF levels. The primary inhibitory effect of the cocktail appears largely attributable to CQ, given the ineffectiveness of TLR2 blockade alone. It is conceivable that plasma components, including albumin, may more substantially attenuate the interaction between target cells and blocker compounds in whole blood compared with PBMCs cultured in 1% FBS, potentially diminishing the efficacy of the blocker cocktail. The outcomes of the whole-blood assays suggest that the TLR cocktail holds promise for exerting an antiinflammatory effect in systemic pneumococcal infection, underscoring the need for further investigations in diverse pneumococcal infection models, beyond those utilized in our study.

Adjunctive antiinflammatory treatment is considered key to improving the outcome of pneumococcal meningitis (2, 3). Accordingly, adjunctive corticosteroids do improve survival in adults with pneumococcal meningitis (22, 68), but unfortunately fail to prevent neurologic complications both in patients and animal models of the disease, as exemplified here by a comparative analysis of cerebral hemorrhages and blood neuronal injury marker concentrations (Figure 3) (21, 35, 69, 70). In our search for additional treatment options, we recently employed a neutralizing TLR2 and -4 specific antibody cocktail in an experimental murine pneumococcal meningitis study. This choice was influenced by the observation that *Tlr2/4^–/–^* mice exhibited reduced inflammation and brain pathology in the early stages of meningitis before antibiotic treatment. However, this treatment failed to significantly affect the experimental disease outcome (14, 35). The more pronounced disease phenotype in *Tlr2/13^–/–^* mice compared with *Tlr2/4^–/–^* mice prompted us to introduce the endosome inhibitor CQ as a TLR13 blocker, combined with the mouse-human cross-reactive neutralizing TLR2 monoclonal antibody T2.5 in our murine meningitis model. T2.5 therapy has been shown to protect mice from antibiotic-treated Gram-negative bacterial hyperinfection pathology when coapplied with an anti-TLR4 antibody (26). CQ, a 4-aminoquinoline, is known as an endocytosis and endosome acidification inhibitor and is used in the treatment of malaria, autoimmune diseases, and cancer (71–73). Moreover, CQ has demonstrated effectiveness as an acute systemic infection pathology and TLR9 blocker in experimental models of polymicrobial sepsis (74, 75). We treated mice undergoing pneumococcal meningitis with a ternary cocktail of ceftriaxone, T2.5, and CQ, which resulted in markedly reduced inflammation, attenuated brain pathology, and consequently improved clinical short-term outcome as compared with mice treated with ceftriaxone alone. In view of these and previous findings such as the resemblance of the disease phenotypes of *Tlr2/13^–/–^* and *C5ar^–/–^* mice lacking the receptor for anaphylatoxin C5a, a key effector molecule of the inflammatory response to pneumococcal CNS infection (35), and adjunctive C5 neutralization having been equally effective (35, 70) as compared with T2.5 and CQ application, we hypothesize that the therapeutic efficacy of an adjunctive treatment regimen is determined by its antiinflammatory potency. This statement is also supported by impressive protective effects through adhesion-promoting neutrophil receptor blockade using a neutralizing anti-CD18 antibody, neutrophil depletion, and neutrophil apoptosis induction (76–78), each eliciting massive neutropenia within the perivascular and subarachnoid space, the site of pneumococcal infection in meningitis, thus blunting inflammatory immune activity therein. It remains to be seen which of the named treatment measures will be able to establish itself clinically. Therein, PRR blockade as a maximally early interference in the potentially fatal immune cascade, namely at the step of pathogen-host interaction, with compounds with known toxicologic profiles such as the T2.5 and CQ cocktail might be a particularly promising candidate.

To date, the glucocorticoid dexamethasone is the drug of choice for adjunctive treatment of pneumococcal meningitis (3, 21, 79). However, dexamethasone negatively influences drug penetration across the blood-brain barrier, resulting in lower drug levels and potentially treatment failure (80). Moreover, dexamethasone broadly suppresses the immune system, albeit with striking cell type– and context-specific activity differences, and thus can hamper the host’s ability to cope with pathogen intrusions (81, 82). Our comparative analysis of adjunctive dexamethasone, combined T2.5/CQ alone, and the triple application of dexamethasone/T2.5/CQ to *S*. *pneumoniae*–infected mice, murine macrophages, and human PBMCs indicated that the dual TLR inhibitor cocktail is significantly more effective than the current gold standard, dexamethasone. Notably, the indistinguishable effects of dexamethasone/T2.5/CQ and T2.5/CQ mix applications suggested a lack of synergism between the antiinflammatory steroid and specific TLR inhibition. However, it also indicated the absence of adverse effects of dexamethasone on T2.5/CQ functions. This suggests the straightforwardness of introducing the application of a triple or quadruple compound combinatory cocktail, such as the one cited here, into pneumococcal meningitis therapy.

By subjecting specific TLR-KO mice to experimental pneumococcal meningitis, we demonstrated the concerted activity of cell-surface TLR2 and endosomal TLR13 as drivers of brain pathology in meningitis. Consequently, combining antimicrobial therapy with TLR2- and endosomal TLR–specific antagonists effectively protected mice from pneumococcal meningitis pathology and significantly inhibited *S*. *pneumoniae*–induced activation of murine macrophages and human PBMCs in which TLR8 substitutes for TLR13. Our results suggest dual TLR blockade as a promising adjunct to antibiotic treatment for pneumococcal meningitis, as its effectiveness surpassed that of dexamethasone in the mouse model. Our observations of a lack or reduction in the effectiveness of dual TLR blockade in whole-blood assays and an i.v. pneumococcal challenge model suggest that this treatment strategy may be ineffective or only partially effective in other pneumococcal infections. To address this specific question, further investigations in additional disease-relevant pneumococcal infection models appear to be necessary.

## Methods

### Sex as a biological variable.

Evidence regarding the role of sex in bacterial meningitis is inconsistent. While most studies did not identify sex as a risk factor for adverse disease progression (83–86), Diederik van de Beek’s group recently reported (a) male sex as an independent risk factor for adverse disease outcomes (87), and (b) higher CSF concentrations of the female sex hormone estradiol were associated with adverse outcomes and increased CSF inflammation (88) — 2 observations that do not align well. In the past 2 decades, we have occasionally used both male and female mice in mouse meningitis studies (e.g., see refs. 14, 89). We found no differences in disease course, brain pathology, and inflammation between females and males. Therefore, we assume that the results of our investigations, even though conducted only in male mice, can be extrapolated to both sexes.

### Animal models of pneumococcal meningitis and sepsis.

A well-established mouse model of pneumococcal meningitis was applied (8, 14). This i.c. infection model offers a clearly defined clinical course. It consistently results in intracranial complications, and since bacteria also enter the bloodstream, it induces systemic complications such as pneumonia. Nevertheless, it is important to acknowledge its limitations, including the fact that it does not replicate the hematogenous route of infection and likely reproduces per continuitatem–driven pneumococcal meningitis.

Briefly, weights and body temperatures of adult (8–12 weeks old) male mice, all on a C57BL/6 genetic background (Charles River), were obtained. Next, mice were clinically examined and scored. Clinical scoring consisted of (a) a beam balancing test, (b) a postural reflex test, and (c) monitoring of the presence of piloerection, seizures, or reduced vigilance. The healthy animal score was set to zero points, while 13 points were attributed to terminally ill animals to be euthanized according to ethics guidelines. Additionally, motor activity was determined by putting mice into the middle of a 42 × 42 cm^2^ open box. The bottom of the box was subdivided into 9 equal-size squares and mice were allowed to explore it for 2 minutes. The number of squares the mice passed through within the observation interval was counted. After clinical examination, bacterial meningitis was induced by i.c. injection of 1 × 10^5^ CFU *S*. *pneumoniae* serotype 2 (D39 strain, NCTC 7466) under short-term anesthesia with isoflurane. Controls were i.c. injected with 10 μL of PBS. All animals that were studied longer than 18 hours p.i. received antibiotic therapy with ceftriaxone (100 mg/kg intraperitoneally [i.p.]), starting at the 18-hour time point. At the end of each experiment, mice were weighed and scored clinically again, and body temperature was measured. After anesthesia with ketamine/xylazine, a catheter was placed into the cisterna magna. CSF was sampled via the catheter to determine leukocyte counts. Subsequently, ICP was monitored using a pressure transducer (Datex-Ohmeda). Moreover, blood samples were drawn by transcardial puncture. Deeply anesthetized mice were perfused with ice-cold PBS containing heparin. Their brains were removed and the separated hemispheres were frozen immediately in tissue-freezing medium (Leica Biosystems) for cryosectioning and histopathological examinations.

For systemic bacterial challenge to model early systemic infection, such as pneumococcal sepsis and treatment, as described above for pneumococcal meningitis, stocks of *S*. *pneumoniae* strain D39 aliquoted at a concentration of 2 × 10^9^ CFU/mL and frozen in the logarithmic growth phase were thawed. They were then incubated upon addition of ceftriaxone to a final concentration of 100 μg/mL for 1 hour at room temperature. Plating 20 μL of the resulting solution on blood agar, followed by overnight incubation at 37°C, confirmed its sterility. At the start of the bacterial incubation period with ceftriaxone, WT C57BL/6 mice were pretreated by i.p. injection of a binary cocktail of the endosomal TLR antagonist CQ and the neutralizing anti-TLR2 antibody T2.5 (in PBS; see dosages below) or PBS solution only (controls). After 60 minutes, 1 × 10^8^ CFU equivalents of ceftriaxone-killed *S*. *pneumoniae* were applied as a 100-μL bolus intravenously. Mice from different groups were sacrificed 2 and 6 hours after the bacterial challenge started, immediately upon which blood was drawn, and spleens and lungs were sampled in a PBS-rinsed fashion. Plasma was aliquoted and frozen. Spleens and right lung lobes were transferred to individual 2 mL polypropylene tubes containing 500 μL lysis buffer (50 mM Hepes [pH 7.6], 150 ml NaCl, 1 mM DTT, 1 mM EDTA [pH 8], 0.5% NP-40, 10% glycerol, 20 mM glycerophosphate, 1 mM Na-orthovanadate, 1 mM EGTA, and protease and phosphatase inhibitor tablets, as indicated by supplier’s instructions, both from Roche). Organs in lysis buffer were immediately cut into small pieces, homogenized, and resulting lysates were incubated for 30 minutes on ice before being centrifuged for 10 minutes at 4°C and 13,000*g*. Supernatants were sampled and frozen. Blood, spleen, and lung specimens described above were evaluated by ELISA and multiplex Luminex assays (R&D Systems).

### Experimental groups.

To model pneumococcal meningitis, 8- to 12-week-old male mice with the following genotypes were used: *Myd88^–/–^* (*n* = 9), *Tlr2/4^–/–^* (*n* = 10), *3d* (*n* = 9), *Tlr3/7/9^–/–^* (*n* = 9), *Tlr2/3/4/7/9^–/–^* (*n* = 10), *3d/Tlr2/4^–/–^* (*n* = 10), *Tlr2/4/13^–/–^* (*n* = 10), and *Tlr2/13^–/–^* (*n* = 10); all strains had a C57BL/6 genetic background (at least 10 backcrosses) and were provided in house. Additionally, C57BL/6 WT (*n* = 18) mice were infected with *S*. *pneumoniae* for comparative analysis. WT mice injected i.c. with PBS served as negative controls (*n* = 8). In order to assess the efficacy of TLR antagonism in the adjunctive treatment of pneumococcal meningitis, WT mice (*n* = 10) were i.p. treated with a cocktail of ceftriaxone (100 mg/kg body weight), a neutralizing anti-TLR2 T2.5 antibody (30 mg/kg body weight; Hycult Biotech, HM1054) (25, 26), and CQ (50 mg/kg body weight; Sigma-Aldrich) (27, 28) 18 hours p.i. and examined 24 hours thereafter (42 hours p.i.). Infected, ceftriaxone-treated (*n* = 10), and ceftriaxone- and mouse IgG1-isotype antibody–treated mice (dissolved in PBS; *n* = 14) constituted the comparison group, while PBS-injected mice served as negative controls (*n* = 4). Applying supplemental groups, we determined whether (a) adjunctive TLR blockade is still effective when executed after the start of antibiotic therapy of pneumococcal meningitis, and (b) the efficacy of TLR antagonism is influenced by dexamethasone, the only adjuvant with proven beneficial effects under clinical conditions (21), which can, however, interfere with drug penetration into the CSF (90). Accordingly, WT mice received T2.5 and CQ 3 hours after antibiotic treatment (*n* = 5). Addressing point b, we compared the effects of a triple combinatory treatment with T2.5, CQ, and dexamethasone (0.5 mg/kg, every 8 hours; *n* = 10) with that of dual therapy with T2.5 plus CQ (*n* = 10) and dexamethasone monotherapy (*n* = 12).

To model acute systemic pneumococcal infection, out of a group of 23 WT mice, 5 were used as comprehensively untreated controls, and 18 were systemically challenged with ceftriaxone-killed pneumococci. Within the latter group, one-half served as controls, while the other half was pretreated with the T2.5 plus CQ cocktail. The 2 groups of bacterially challenged, pretreated, and control mice were further divided into 2 groups and sacrificed at 2 hours (*n* = 5 each) or 6 hours (*n* = 4 each) after the start of the bacterial suspension challenge for specimen sampling.

### Determination of bacterial titers in blood and brain.

Cerebella were dissected and homogenized in sterile saline. Blood samples and cerebellar homogenates were diluted serially in sterile saline, plated on blood agar plates, cultured for 24 hours at 37°C with 5% CO_2_, and colonies thereon were counted.

### Analysis of cerebral bleeding.

Mouse brains were cut in a frontal plane into 10-μm-thick sections. Beginning from the anterior parts of the lateral ventricles, 10 serial sections were photographed with a digital camera at 0.3-mm intervals throughout the ventricle system. Hemorrhagic spots were counted and total bleeding area on each slice was determined using ImageJ software (NIH).

### Measurement of IL-6, IL-1β, TNF, and additional cyto- and chemokines in brain, lung, and spleen homogenates, blood plasma, and whole blood and cell culture supernatants.

Specific cellular inflammatory mediator concentrations in specimens and experimental cultures using murine and human cellular materials were determined by ELISA and bead-based multiplexing (R&D Systems) following the manufacturer’s instructions.

### Cell culture experiments.

Mouse BMDMs were prepared from bone marrow cells isolated from femurs. Briefly, bones were flushed with Hanks’ balanced salt solution and the cell suspension was forced through a 70-μm mesh. Collected cells were resuspended in complete macrophage medium consisting of Dulbecco’s modified Eagle’s medium (DMEM), 50 ng/mL recombinant macrophage colony–stimulating factor (M-CSF, Peprotech), 10% FBS, 10 mM HEPES, 10 mM L-glutamine, and 10 μg/mL penicillin/streptomycin (P/S) (all from Sigma-Aldrich) and cultured at 37°C in 5% CO_2_. After 1 day, the floating cells were replated in fresh culture medium and on day 4 the medium was replaced. After 7 days, virtually 100% of the cells expressed the macrophage markers CD11b and F4/80, according to punctual flow cytometric analyses. The culture medium was replaced with macrophage medium containing 1% FBS, but lacking M-CSF as well as P/S. Again, 24 hours later, cells were exposed to *S*. *pneumoniae* (1 × 10^6^ CFU/mL; MOI = 10) and treated with ceftriaxone (100 μg/mL) starting 4 hours p.i. Antibody T2.5 (25 μg/mL (25, 26) and/or CQ (20 μg/mL) (27, 28) were added to the cell culture medium 2 hours before, simultaneously with, or 2 hours after the antibiotic administration. Controls received the vehicle solution of *S*. *pneumoniae* – specifically 0.5% Todd-Hewitt broth with 2% yeast extract dissolved in PBS (THY). Lactate dehydrogenase (LDH) and IL-6 release was determined as indicators of macrophage cell death and activation, respectively, in cell culture supernatants collected 24 hours p.i. (Biovision Inc and R&D Systems).

Venous blood samples were collected from healthy and drug-free volunteers in EDTA-containing Microvette tubes. PBMCs were prepared by density gradient centrifugation of EDTA blood over Histopaque 1.077 for 30 minutes and 900*g* at room temperature after 1:2 (v/v) dilutions in DMEM. Mononuclear cells forming a cell layer above Histopaque 1.077 were collected and added to a fresh tube. After hypotonic erythrocyte lysis and washing thrice with PBS, PBMCs were resuspended in DMEM containing 1% FBS and pipetted into individual wells of 96-well cell culture plates (200,000 cells/well). PBMCs and BMDMs were challenged in identical manners except for the treatment with dexamethasone (5 μg/mL) and the TLR8 antagonist Cu-Cpt9a (10 μg/mL; Tocris) (66). Both compounds were used in PBMC culture exclusively, given that the former compound is the pneumococcal meningitis adjunctive therapy gold standard and application of the latter evaluated pan-endosomal TLR inhibitory CQ specificity in our experimental setting.

For whole-blood assays, blood samples (10 mL) were collected from age- and sex-matched healthy volunteers (*n* = 20). The blood was collected using heparin-containing S-Monovettes (Sarstedt) and mixed in a 1:1 ratio with serum-free RPMI 1640. Subsequently, aliquots of 180 μL were dispensed into individual wells of a 96-well polystyrene plate (Nunc, Thermo Fisher Scientific) and incubated under standard culture conditions (37°C, humidity-saturated atmosphere, 5% CO_2_). Specific wells were pretreated with T2.5, CQ, or both substances (dosages as indicated above; in 10 μL) and inoculated with 1 × 10^8^ CFU equivalents/mL (in 10 μL) of ceftriaxone-killed *S*. *pneumoniae*. After 6 hours, supernatants were collected, centrifuged, and stored frozen for the measurement of inflammatory mediator concentrations. Alternatively, samples were infected with 1 × 10^6^ CFU/mL viable *S*. *pneumoniae*. After 4 hours, ceftriaxone alone or in combination with the above-mentioned antiinflammatory compounds was added, and the incubation continued for an additional 20 hours, after which supernatants were processed as described above.

### Statistics.

The principal statistical test was 1-way analysis of variance (ANOVA) and subsequent Tukey’s post hoc test. A *P* value of less than 0.05 was considered significant. In case of group size smaller than or equal to 5 in all groups, the data are displayed as dot plots along with mean ± SD. For larger sample sizes, data are illustrated as box-and-whisker plots, with lines in boxes showing median and bounds of the boxes showing the range between 25% and 75% percentiles, and whiskers indicating minimal and maximal values.

### Study approval.

This study was carried out in accordance with the recommendations in the NIH *Guide for the Care and Use of Laboratory Animals* (National Academies Press, 2011) and with the German Animal Protection Act. The pneumococcal meningitis study protocol was approved by the Committee on the Ethics of Animal Experiments of the Government of Upper Bavaria, Germany (permit numbers 55.2-1-54-2531-32-04, 54-2531-47-08, and 54-2531-143-12). Pretreatment and systemic challenge for not more than 6 hours with antibiotic-killed bacteria experiments were performed upon approval of a respective mouse experimentation project application to the North Rhine-Westphalia State Office for Nature, Environment and Consumer Protection, Recklinghausen, Germany (file reference 81-02.04.2020.A499). Venous blood samples were collected from healthy and drug-free volunteers after obtaining written informed consent (ethics approval number 105-16). Blood samples for whole-blood assays were collected after obtaining written informed consent and Ethics Committee (LMU Munich) approval (ethics vote 18-717).

### Data availability.

The authors confirm that the underlying data associated with the main manuscript and supplemental material are provided in a single Supporting Data Values XLS file in the supplemental material.

## Author contributions

SDS and IM performed experiments and drafted the manuscript. SH provided bacteria and comments on the manuscript. BA performed analyses. SV, HWP, and MK participated in its design and provided comments on the manuscript. HI, VM, and JB contributed to analyses and illustrated results. CJK, JB, and VM provided the KO mouse strains. UK and CJK designed and performed experiments and drafted the manuscript. All authors read and approved the final manuscript.

## Supplementary Material

Supplemental data

Supporting data values

## Version 1 02/15/2024

In-Press Preview

## Version 2 03/22/2024

Electronic publication
