# Supplementary material for: Combining antibiotic with anti-TLR2/TLR13 therapy prevents brain pathology in pneumococcal meningitis
Source: JCI Insight. 2024 Feb 15;9(6):e165737. doi: 10.1172/jci.insight.165737 (PMC10972620; doi:10.1172/jci.insight.165737)
Supplement: Supplemental data [file jciinsight-9-165737-s006.pdf]

Supplemental Figures

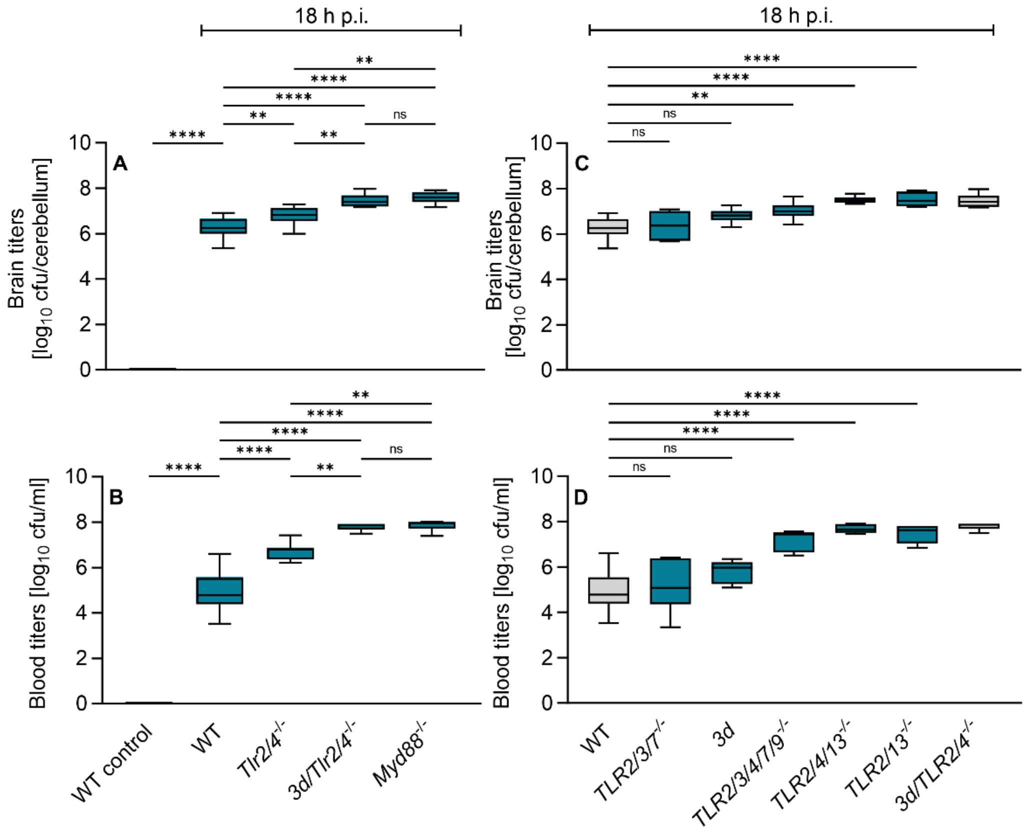

Supplemental Figure 1: Illustration of bacterial loads in pneumococcal meningitic mice of the specific genotypes indicated, with integer values on the y-axis as an alternative presentation to the data shown in Figures 1 and 2

Diagrams comparatively depict bacterial loads in pneumococcal meningitic mice 18 hours post-infection in the indicated compartments, already illustrated by Figures 1 and 2. The continuity of the y-axes provides a more comprehensive view of the extent of the infection compared to the presentation in Figures 1 and 2 (left and right panels, respectively), emphasizing differences in infected mice for which the y-axes are graphically interrupted. Data are given as median with 25%-75% percentile range, min and max. The data from the WT and *3d/Tlr2/4*$^{-/-}$ groups (gray boxes in C and D) correspond to those in A and B, respectively. The number of samples in individual experiments may be lower if mice had to be euthanized before the end of the

experiment or technical issues appeared (for detailed information see “Supporting data values” file). Statistical test was one way ANOVA and subsequent Tukey post-hoc tests (****, $P < 0.001$; ***, $P < 0.001$; **, $P < 0.01$; *, $P < 0.05$; ns, not significant).

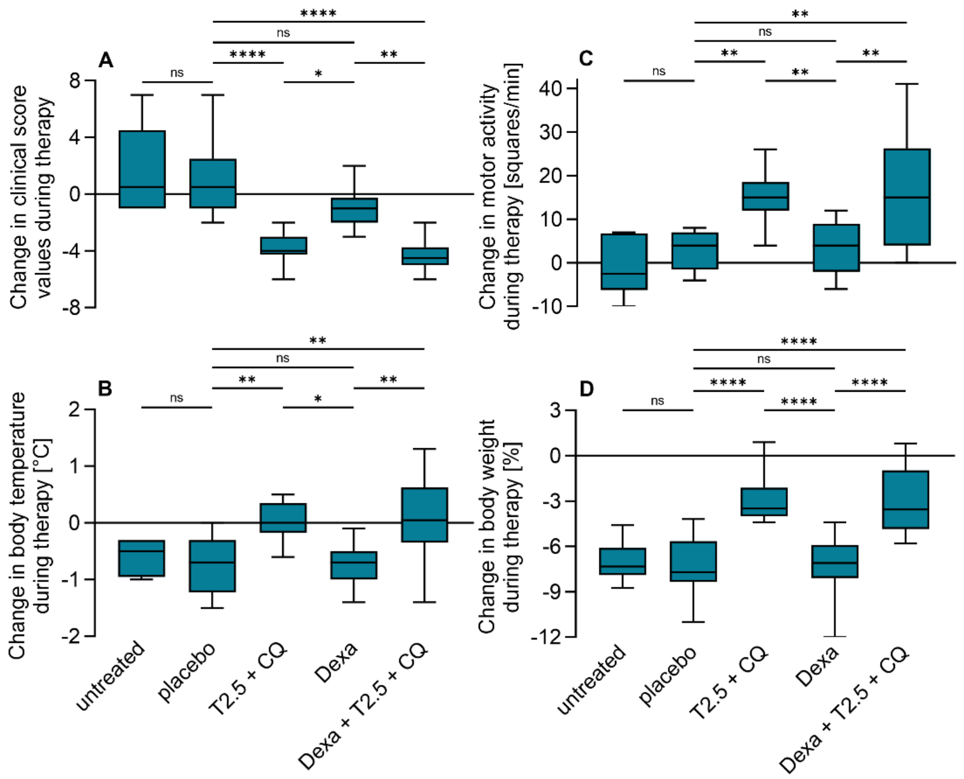

Supplemental Figure 2: Presentation of the changes in clinical measurement parameters resulting from the indicated therapeutic interventions in WT mice with pneumococcal meningitis.

The depicted values represent the changes in four clinical measurements, namely clinical score values, motor activity, body temperature, and body weight, over time, from the initiation of therapy to the end of the experiment in mice with pneumococcal meningitis. In all depicted experimental groups, mice were treated with ceftriaxone 18 hours after infection. In the two control groups, mice either received no additional treatment (n=10) or a placebo (n=14). As adjunctive treatment measures, either the neutralizing anti-TLR2 antibody T2.5 and chloroquine (CQ) (n=10), dexamethasone (DEXA) (n=12), or T2.5, CQ, and DEXA combined (n=10) were administered. Data are given as median with 25%-75% percentile range, min and max. The number of samples in individual experiments may be lower if mice had to be euthanized before the end of the experiment or if technical issues appeared (for detailed information see

“Supporting data values” file). Statistical test was one way ANOVA and subsequent Tukey post-hoc tests (****, $P < 0.001$; ***, $P < 0.001$; **, $P < 0.01$; *, $P < 0.05$; ns, not significant).

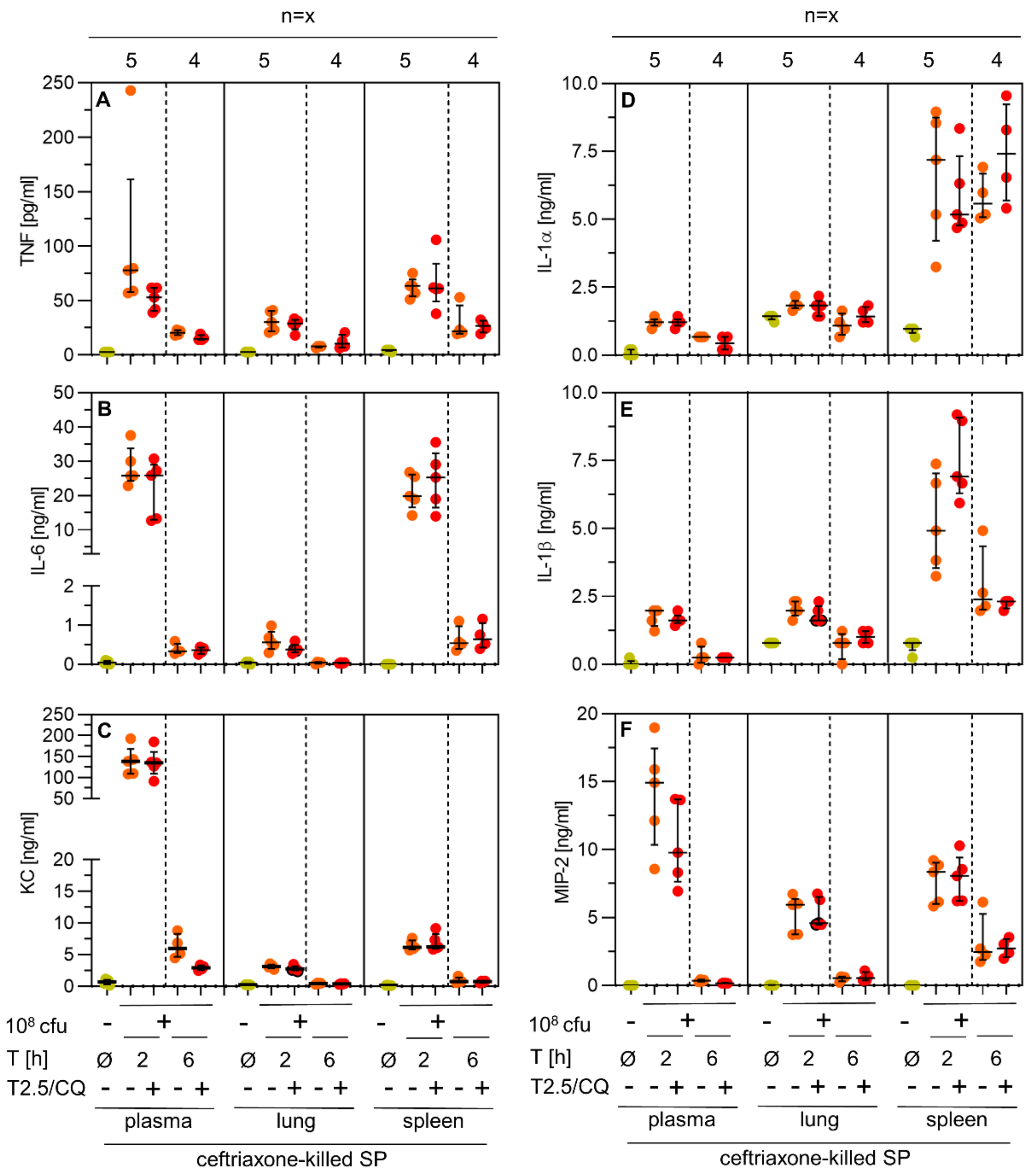

Supplemental Figure 3: Lack of therapeutic effectiveness was observed with the dual T2.5/CQ cocktail administered 1 hour prior to systemic challenge with ceftriaxone-killed *S. pneumoniae* in mice.

Mice, organized into experimental groups of the indicated sizes, were either left unchallenged or systemically challenged with ceftriaxone-killed *S. pneumoniae* after intraperitoneal (i.p.) administration of either the placebo PBS or a dual cocktail containing neutralizing anti-TLR2 mAb (T2.5) and chloroquine (CQ) 1 hour prior. At either 2 (n=5 each) or 6 (n=4 each) hours after the bacterial challenge, blood plasma,

lung, and spleen specimens were sampled, processed through centrifugation or homogenization in lysis buffer, and the resulting solutions were analyzed by multiplex ELISA. Individual sample values, as well as medians with interquartile ranges, are depicted.

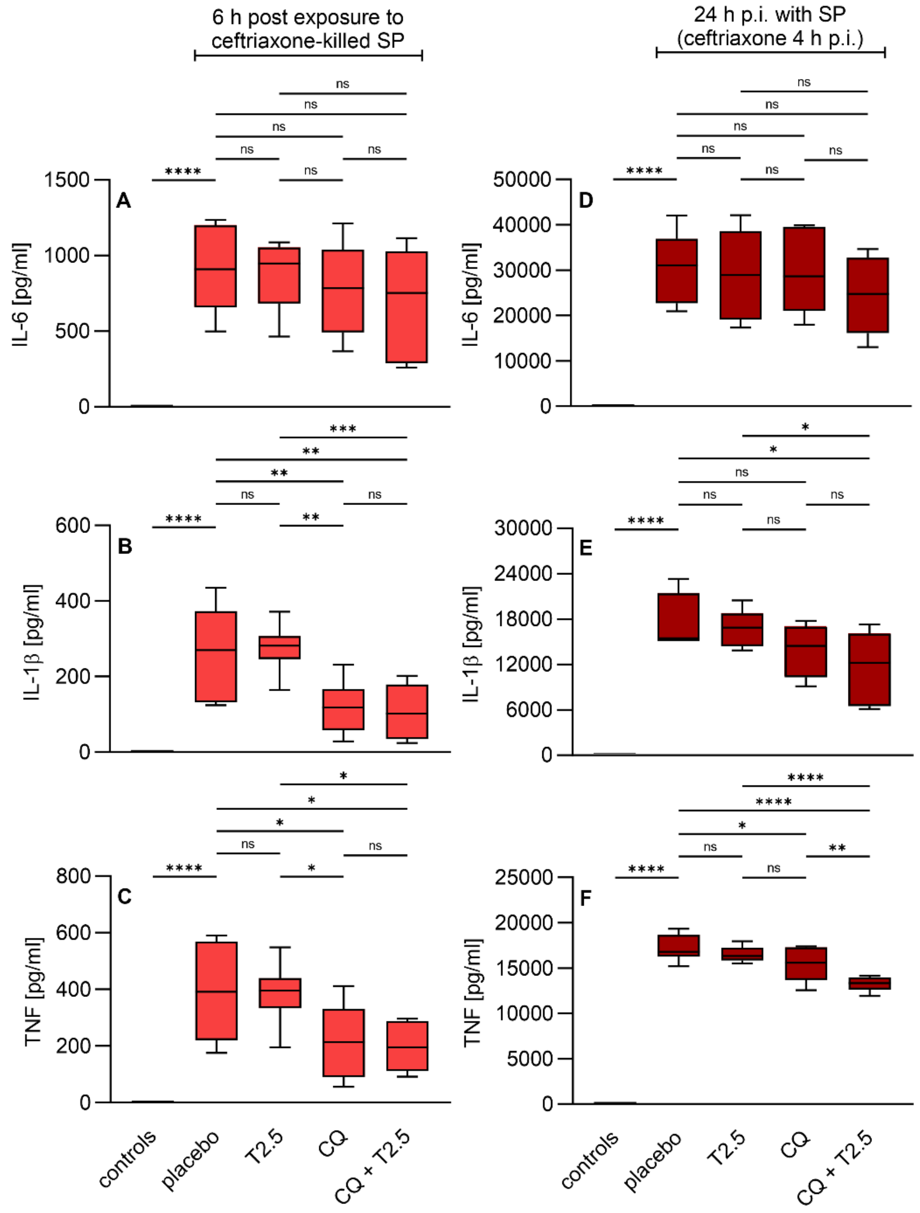

Supplemental Figure 4: The immune inhibitory effect of the dual T2.5/CQ cocktail on *S. pneumoniae*-induced cytokine release was significantly less pronounced when applied to human whole blood compared to isolated PBMC.

Aliquots of heparinized whole blood from a healthy human donor (mixed in a 1:1 ratio with serum-free RPMI1640) were cultured under standard cell culture conditions in a 96-well plate format, with measurements taken in quadruplicates two times. The samples were either challenged with ceftriaxone-killed *S. pneumoniae* or infected with viable *S. pneumoniae* (left panel, bright red, and right panel, dark red, respectively, except for controls). In the former case, cells were treated as indicated 1 hour prior to

bacterial challenge, while infected cultures were treated concomitantly with the antibiotic ceftriaxone at 4 hours post-infection (p. i.). Supernatants were collected at 6 hours and 24 hours after challenge, respectively. Data are from 2 independent experiments conducted in quadruplicates and depicted as median with 25%-75% percentile range, min and max. Statistical test was one way ANOVA and subsequent Tukey post-hoc tests (****, $P < 0.001$; ***, $P < 0.001$; **, $P < 0.01$; *, $P < 0.05$; ns, not significant; placebo, 0.1% DMSO in PBS; T2.5, neutralizing anti-TLR2 mAb; CQ, chloroquine).
